# Elevated plasma GDF15 combined with FGF21 suggests mitochondrial dysfunction in a subgroup of anorexia nervosa patients

**DOI:** 10.1038/s41398-025-03425-0

**Published:** 2025-06-25

**Authors:** Jingjing Xu, Ruyue Zhang, Vincent Millischer, Miranda Stiernborg, Claire E. Tume, Sara Mehdinia, Peter Barker, Zeynep Yilmaz, Vanessa F. Gonçalves, Catharina Lavebratt, Mikael Landén, Stephen O’Rahilly, Cynthia M. Bulik, Ida AK Nilsson

**Affiliations:** 1 Department of Molecular Medicine and Surgery, Karolinska Institutet, Stockholm, Sweden; 2 Center for Molecular Medicine, Karolinska University Hospital, Stockholm, Sweden; 3 Department of Medical Epidemiology and Biostatistics, Karolinska Institutet, Stockholm, Sweden; 4 Department of Genetics, University of North Carolina at Chapel Hill, Chapel Hill, NC USA; 5 Department of Psychiatry and Psychotherapy, Medical University of Vienna, Vienna, Austria; 6 Comprehensive Center for Clinical Neuroscience and Mental Health, Medical University of Vienna, Vienna, Austria; 7 NIHR Cambridge Core Biochemical Assay Laboratory, Cambridge University Hospitals NHS Foundation Trust, Cambridge, UK; 8 National Centre for Register-based Research, Aarhus University, Aarhus, Denmark; 9 Department of Psychiatry, University of North Carolina at Chapel Hill, Chapel Hill, NC USA; 10 Department of Biomedicine, Aarhus University, Aarhus, Denmark; 11 Molecular Brain Sciences, Centre for Addiction and Mental Health, Toronto, ON Canada; 12 Department of Psychiatry, University of Toronto, Toronto, ON Canada; 13 Department of Pharmacology & Toxicology, University of Toronto, Toronto, ON Canada; 14 Department of Psychiatry and Neurochemistry, Institute of Neuroscience and Physiology, the Sahlgrenska Academy at the University of Gothenburg, Gothenburg, Sweden; 15 MRC Metabolic Diseases Unit, Institute of Metabolic Science, University of Cambridge, Addenbrookes Hospital, Cambridge, UK; 16 Centre for Eating Disorders Innovation, Department of Medical Epidemiology and Biostatistics, Karolinska Institutet, Stockholm, Sweden; 17 Department of Nutrition, University of North Carolina at Chapel Hill, Chapel Hill, NC USA

**Keywords:** Psychiatric disorders, Molecular neuroscience

## Abstract

Growth and differentiation factor 15 (GDF15) is a significant player in cellular stress and energy homeostasis. GDF15 is elevated in cancer cachexia, chemotherapy-induced anorexia, hyperemesis gravidarum, and mitochondrial disorders. Here we analyze GDF15 in anorexia nervosa (AN), a psychiatric disorder characterized by low weight and persistent restriction of food intake. While no significant difference in plasma GDF15 concentration was seen across the three included groups; active AN, recovered AN, and healthy controls, a subgroup of study participants with high GDF15 plasma was noted to a significantly higher extent in the AN groups. Sparse partial least squares discriminant analysis (sPLS-DA) identified six markers related to inflammatory processes or cellular stress from a set of 74 markers that distinguished AN with high GDF15 from the rest, with fibroblast growth factor 21 (FGF21) being the most important contributor. Moreover, FGF21 plasma concentration was significantly higher in the group with high GDF15, suggesting an involvement of mitochondrial dysfunction. In fact, mitochondrial polygenic risk score (PRS) was significantly associated with AN risk in a large AN case-control cohort. In line with this, we also report elevated liver expression of GDF15 in the *anx/anx* mouse displaying anorexia associated with mitochondrial dysfunction. We conclude that mitochondrial dysfunction should be further explored in AN. Clinical trials of GDF15 immunoneutralization in patients with AN and high levels of GDF15 are worthy of consideration.

## Introduction

Anorexia, i.e., low appetite and/or food intake, accompanies several conditions including cachexia of cancer and inflammatory disorders, chemotherapy-induced anorexia, nausea and vomiting of pregnancy, hyperemesis gravidarum, and the psychiatric disorder anorexia nervosa (AN). The potent appetite-inhibiting cytokine growth and differentiation factor 15 (GDF15), previously known as macrophage inhibitory cytokine-1 (MIC-1) [1], has been reported as elevated in all these conditions [1–4], although to our knowledge, only two studies with small sample sizes (*n* = 16/20) have explored GDF15 in AN [5, 6]. This cytokine, originally identified as a product of activated macrophages, is now well established as a cellular and nutritional stress-induced hormone that acts on the hindbrain to co-ordinate an “illness response” including anorexia, nausea, vomiting, physical inactivity, and the activation of neuroendocrine stress responses [3, 7]. It is increased in obesity [8, 9] and overfeeding appears to activate a stress response in the liver, the major source for systemic elevations of this hormone [10]. Nevertheless, animals and humans lacking GDF15 are not obese [11], while overexpressing GDF15 in mice results in reduced body weight [12]. Additionally, data indicate that GDF15 causes anorexia by inducing nausea and/or by engaging emetic neurocircuitry in the area postrema and nucleus tractus solitarius [1]. The receptor for GDF15, glial cell-derived neurotrophic factor receptor alpha-like (GFRAL), is exclusively expressed in these two brain regions [13–15]. Activation of GFRAL reduces food intake and body weight in animal models [12]. Inhibition of GDF15 in mice and humans reverses cancer cachexia [16, 17], including reducing anorexia and emesis [4], restoring muscle function as well as physical performance [18]. Lastly, intense exercise has been shown to increase circulating GDF15 in humans [19, 20].

The two small studies that previously explored GDF15 in females with active AN reported increased serum levels [5, 6]. Two months of nutritional therapy leading to partial weight recovery significantly decreased GDF15 in AN, even if the concentrations remained significantly higher in AN compared with healthy controls [5]. AN is a severe mental disorder with a high mortality rate, characterized by persistent restriction of food intake, fear of gaining weight, and concerns about body weight and shape [21]. The twin-based heritability of AN is estimated to be 50–60% and genome-wide association studies (GWAS) have to date identified eight variants in the genome significantly associated with AN risk [22]. A puzzling part of AN is a seemingly paradoxical response to negative energy balance, wherein patients actively strive to consistently expend more energy than they consume, resulting in continued loss of body weight and low energy stores. Loss of weight promotes changes in hormonal and other signals from adipose tissue (e.g., reduced leptin) and gut to instruct the brain to increase appetite and reduce energy expenditure, thus restoring energy stores and ensuring survival [23]. However, individuals with AN reside in persistent severe starvation, emaciation, and negative energy balance often for many years, and frequently revert to that state even after therapeutic weight restoration as if their biology defends their low weight state. The definition of the molecular underpinnings of anorexia, i.e., the loss or absence of appetite, might therefore define drug targets supporting renourishment and weight gain in AN.

The *anx/anx* mouse is a genetically spontaneously arisen model mimicking central aspects of AN; starvation and underweight [24]. The mouse eats less compared with its healthy littermates despite unrestricted access to food, subsequently becomes emaciated, and dies prematurely around three weeks of age. A range of deviations in hypothalamic neuropeptidergic and neurotransmitter systems have been documented in the *anx/anx* mouse [25]. The mouse exhibits a dysfunction in complex I of the oxidative phosphorylation system of the mitochondria present before the anorectic phenotype develops [26]. A small study in humans also described a similar mitochondrial dysfunction in leukocytes from patients with AN [27]. Interestingly, GDF15 is reported to be elevated in other animal models of mitochondrial dysfunction [28, 29], and human mitochondrial disorders [30] and has therefore been proposed as a biomarker to screen for mitochondrial disorders [31].

Based on our hypothesis that elevated plasma GDF15 in AN disrupts energy homeostatic regulation, because of mitochondrial dysfunction, we evaluated plasma levels of GDF15 in the to date largest cohort of women with active (*n* = 70) or recovered from AN (*n* = 89) and normal-weight women with no histories of eating disorders (*n* = 72). We explored potential contributors to the difference of GDF15 in the two AN groups by feature selection using a large set of circulating proteins many of but not all of which are involved in inflammation. Given the genetic basis of AN [22, 32–34], we further evaluate the association between mitochondrial function polygenic risk score (PRS), a personalized genetic risk score based on single nucleotide polymorphisms (SNPs) within the gene regions associated with mitochondrial function, and the risk of AN in a larger AN case-control cohort. Furthermore, we analyze correlations between plasma GDF15 and the adipose-derived hormone leptin and perform stratified analyses of typical AN characteristic such as purging behaviors. Lastly, we evaluated expression of GDF15 and an associated marker in the liver of the anorectic *anx/anx* mouse with established mitochondrial dysfunction.

## Materials and methods

### Study participants and design

The study participants were identified from the Swedish sample of the Anorexia Nervosa Genetics Initiative (ANGI-SE, including in total 4118 AN cases and 4035 controls). Details on the recruitment procedure for ANGI-SE have been described previously [35].

For the subcohort that provided plasma, the **AN group** comprised women at least 18 years of age at recruitment, meeting DSM-IV criteria for AN [36] except for amenorrhea, and a minimum of one year since AN onset (*n* = 70). For those **recovered from AN**, the inclusion criteria were a history of DSM-IV AN followed by weight restoration (BMI > 20 kg/m^2^) and no eating disorder behaviors for at least a year (AN-REC, *n* = 89). The age-matched normal-weight **controls** reported no history of disordered-eating behavior (CTRL, *n* = 72).

Exploratory analyses of the two AN groups were done investigating subtypes: defined by episode(s) of binge eating with loss of control as AN with binge eating (AN-B, *n* = 26, AN-REC-B *n* = 59) compared to the complete absence of such episodes as AN without binge eating (AN-noB, *n* = 37, AN-REC-noB, *n* = 24); episodes of laxative use (AN-LAX, *n* = 11, AN-REC-LAX, *n* = 18) compared to without laxative use (AN-noLAX, *n* = 58, AN-REC-noLAX, *n* = 69); documented self-induced vomiting (AN-VOM, *n* = 29, AN-REC-VOM, *n* = 46) compared to without self-induced vomiting (AN-noVOM, *n* = 40, AN-REC-noVOM, *n* = 41); reported episodes with use of diuretics (AN-DIU, *n* = 6, AN-REC-DIU, *n* = 5) compared to without diuretics use (AN-noDIU, *n* = 43, AN-REC-noDIU, *n* = 55); and reported compensatory exercise (AN-EXE, *n* = 41, AN-REC-EXE, *n* = 54) compared to without compensatory exercise (AN-noEXE, *n* = 5, AN-REC-noEXE, *n* = 7). See Table 1 for detailed characteristics of the study participants. The ANGI-SE study was approved by the Regional Ethics Review Board in Stockholm. All participants gave written informed consent. When applicable the investigators were blinded to group allocation when conducting the analyses.

**Table 1:** Sex, age, BMI, years since AN onset, and eating disorder behaviors of the study participants by group, significant differences are marked in bold and with a AN vs CTRL, b AN vs AN-REC.

Characteristics	AN	AN-REC	CTRL
n	70	89	72
Women (%)	100	100	100
Age at sample (years)	26	26	26
(median [IQR])	(24.0–31.0)	(24.0–31.0)	(24.0–31.0)
Age of first AN onset (years)	16	16	
(median [IQR])	(14.0–19.0)	(14.0–19.0)	
BMI at sample (kg/m^2^)	**16**	22	23
(median [IQR])	**(15.2**–**16.7)**^**a,b**^	(20.8–24.5)	(22.0–26.0)
Minimum BMI during AN (kg/m^2^)	**13.7**	16.7	
(median [IQR])	**(12.2**–**14.4)**^**b**^	(14.9–17.9)	
Years since AN onset	10	10	
(median [IQR])	(6.0–14.0)	(6.0–15.0)	
Length of amenorrhea (years)	**4.5**	1.5	
(median [IQR])	**(2.0**–**9.0)**^**b**^	(0.5–3.0)	
Subtype (n [%])			
with Binge-eating	26 (41.3%)	59 (71.1%)	
without Binge-eating	37 (58.7%)	24 (28.9%)	
Laxative use;			
Never	58 (84.1%)	69 (79.3%)	
Ever	11 (15.9%)	18 (20.7%)	
Self-induced vomiting;			
Never	40 (58%)	41 (47.1%)	
Ever	29 (42%)	46 (52.9%)	
Diuretic;			
Never	43 (87.8%)	55 (91.7%)	
Ever	6 (12.2%)	5 (8.3%)	
Compensatory exercise;			
Never	5 (10.9%)	7 (11.5%)	
Ever	41 (89.1%)	54 (88.5%)	

### Blood sampling

Blood samples were collected in EDTA tubes at a hospital near the participant’s home address, sent to Karolinska Institutet Biobank with overnight mail, and processed upon arrival. After centrifugation, plasma samples were stored at −80 °C. All samples were exposed to two freeze-thaw cycles prior to analyzing GDF15 concentrations.

### Plasma GDF15 concentrations

We measured GDF15 in plasma by quantitative sandwich enzyme immunoassay (*n* = 6 plates, R&D Systems Quantikine® ELISA assay, Minneapolis, MN). In brief, plasma samples were diluted fourfold. Samples and standards were pipetted in duplicates into the wells and any GDF15 present was bound by the immobilized antibody on the bottom of the wells. After adding horseradish peroxidase (HRP)-conjugated polyclonal antibody recognizing GDF15 followed by a substrate solution, color developed in proportion to the amount of GDF15 bound in the initial step. The absorbance was measured on a plate reader (Spectramax Plus, Molecular Devices, San Jose, CA).

A very common histidine to aspartate variant at position 202 of the GDF15 pro-peptide in humans substantially affects its measurement by the R&D assay [37]. We here corrected the concentration of GDF15 based on this variant as described by Karusheva et al. [37], utilizing the genetic data collected in ANGI-SE [35]. All statistical analyses were conducted on the genotype-corrected concentrations.

### Plasma leptin

We measured leptin in plasma by an in-house quantitative sandwich time-resolved fluorescence (DELFIA^®^) immunoassay (*n* = 7 plates) using antibodies and standards from R&D Systems, Minneapolis, MN, and Europium-labelled Streptavidin, buffers and enhancement solution from Revvity, Waltham, MA). In brief, the microtiter plate was coated with a monoclonal anti-leptin capture antibody. Standards, controls, and samples were added to the plate in duplicate. After incubation and washing, a biotinylated polyclonal anti-leptin detection antibody was added to the plate. After incubation and washing, Europium-labelled Streptavidin was added. After another wash Enhancement Solution was added to the wells which allowed the fluorescence to be generated in the wells when illuminated. The intensity of the time-resolved fluorescence is directly proportional to the concentration of leptin in the standards, controls, and samples. The leptin analysis was undertaken on the AutoDELFIA^®^ analyzer (Revvity, Waltham, MA).

We analyzed the correlation between plasma leptin and plasma GDF15.

### Plasma markers related to inflammatory processes and cellular stress

In a recent paper from our group exploring the Olink Proteomics inflammation panel (Uppsala, Sweden) utilizing the same cohort as here, we quantified the plasma concentrations of 74 immune activation markers [38]. The log2-transformed normalized expression data of these markers, together with leptin and GDF15, were used to select the most discriminative features that distinguished individuals with high GDF15 (>800 pg/ml) from the remaining individuals (<800 pg/ml) by sparse partial least squares discriminant analysis (sPLS-DA).

### Mitochondrial polygenic risk score

We constructed a PRS of mitochondrial genes for each individual in ANGI-SE, which aggregates genetic risk for each individual from single nucleotide polymorphisms (SNPs) located within the regions of mitochondrial genes, using PRS-continued shrinkage (CS) [39]. Mitochondrial genes (*n* = 1136) were obtained from MitoCarta 3.0 database [40]. MitoCarta is an inventory of human and mouse genes with strong evidence for mitochondrial function. Genotype quality control of the ANGI-SE sample has been described in detail previously [22, 41] which resulted in 4054 AN cases and 3922 controls. AN GWAS summary statistics [22] without Swedish participants were used as the discovery dataset providing effect sizes and standard errors for PRS-CS [39] to get posterior SNP effect size estimates. We included common non-ambiguous SNPs within the listed regions with genes related to mitochondrial function (Supplementary Table 1) with high imputation quality (INFO ≥ 0.8) and minor allele frequency (MAF) ≥ 0.01 in the PRS calculation, resulting in a subset of 2475 SNPs. The 1000 Genomes Project Phase 3 EUR reference was used as linkage disequilibrium reference panel. After applying the posterior SNP effect size estimates from PRS-CS across chromosomes to get individual PRS via PLINK 1.9 [42], these scores were then standardized in R (version 4.3.2) [43] for further regression analyses.

### Animals

Experiments involving animals followed the procedures approved by the ethical committee (Stockholms norra djurförsöksetiska nämnd) and were designed to minimize suffering. An intercross was set up using heterozygous *anx* breeding pairs (B6C3Fe–a/a–anx A/+a) acquired from the Jackson Laboratory (Bar Harbor, ME). All mice were genotyped using simple sequence length polymorphism markers mapped to the subchromosomal region, where the *anx* mutation is located. Phenotypic characterization was based on body weight. The mice were housed in ventilated cages at 25 °C in an animal room with a 12 h light-dark cycle (lights on at 7:00 AM) and with unrestricted access to the mother’s milk. Pups were sacrificed by decapitation between postnatal days (P) 19–21, with the day of delivery considered P1. The liver was rapidly dissected and frozen in ice-cold isopentane. We included seven liver samples from *anx/anx* and six from healthy wild-type siblings (+/+ or *anx*/+), all females. All analyses were conducted blinded of genotype.

### Quantitative polymerase chain reaction (qPCR)

Liver tissues were homogenized with ZR BashingBead Lysis Tubes (S6012–50, Zymo Research, Irvine, USA) in Trizol Reagent (15596018, Invitrogen, Carlsbad, USA). Total RNA was extracted using Direct-zol™ RNA Miniprep (R2050, Zymo Research) according to the manufacturer’s instructions. DNase-treated RNA was reverse transcribed to cDNA using SuperScript™ III First-Strand Synthesis System (18080051, Invitrogen). Reactions were performed in three replicates with primers for GDF15 (5′-AACCCCTGGTCTGGGGATAC-3′ (forward); 5′-CATGTCGCTTGTGTCCTTTCAG-3′(reverse)), FGF21 (5′-CCTTGAAGCCAGGGGTCATT-3′ (forward); 5′-GGATCAAAGTGAGGCGATCCA-3′(reverse)), and GAPDH (5′-ACCCTTAAGAGGGATGCTGC-3′ (forward); 5′-CCCAATACGGCCAAATCCGT-3′(reverse)) using iTaq™ Universal SYBR® Green PCR kit (1725124, Bio-Rad, Hercules, USA) on a QuantStudio™ 6 Real-Time PCR Instrument (ThermoFisher, Waltham, USA). The PCR consists of 95 °C for 10 min, 95 °C 15 s −> 60 °C for 60 s for 40 cycles, and 72 °C for 5 min. The measured GDF15 transcript abundance was normalized to GAPDH using the delta-delta Ct method.

### Statistical analyses

Group differences in plasma concentrations of GDF15 and leptin in AN, AN-REC, and CTRL were analyzed using the Kruskal-Wallis test since the concentrations were not normally distributed. This was followed by post-hoc Dunn’s test with Bonferroni correction to evaluate pairwise comparisons. ANCOVA was used to control for the effect of BMI when comparing the leptin levels of AN and AN-REC, followed by Tukey’s test. Associations among GDF15, body mass index (BMI), leptin, and immune activation markers were assessed using Spearman correlation. Principal component analysis (PCA) was used to identify multi-dimensional outliers in the immune activation markers dataset. sPLS-DA was then used to discriminate the groups of individuals with high GDF15 (>800 pg/ml) and the rest of the individuals (<800 pg/ml). A five-fold, ten-repeat cross-validation procedure was used to select the number of components and variables of the sPLS-DA model, and balanced error rate to evaluate model performance. The sPLS-DA results were supported by other feature selection techniques such as Lasso regression (data not shown). Group differences in plasma concentrations of GDF15 in AN-B vs. AN-noB, AN-REC-B vs. AN-REC-noB, AN-LAX vs. AN-noLAX, AN-REC-LAX vs. AN-REC-noLAX, AN-VOM vs. AN-noVOM, AN-REC-VOM vs AN-REC-noVOM, AN-DIU vs AN-noDIU, AN-REC-DIU vs AN-REC-noDIU, AN-EXE vs AN-noEXE, AN-REC-EXE vs AN-REC-noEXE were tested using the nonparametric Mann-Whitney U test, and effect sizes were measured by Cohen’s d. To evaluate the associations between high plasma GDF15 and AN diagnosis and behaviors, the Chi-square test was used, whereas Fisher’s exact test was used when at least one cell of the contingency tables contained values below 5. Phi Coefficient (φ) and odds ratio (OR) were reported as effect size estimates for the Chi-square and Fisher’s exact test, respectively. Logistics regressions were conducted to examine the associations between AN risk and mitochondrial function PRS, including the first ten genetic principal components (PCs) as covariates. The ANGI-SE cohort was then divided into quartiles based on the mitochondrial PRS level (i.e., low, medium-low, medium-high, and high), with the lowest quartile serving as the reference group. The risk of AN was then compared across these mitochondrial PRS quartiles using logistic regressions, adjusting for the first ten genetic PCs. Finally, linear regression was conducted to test the association between GDF15 and FGF21 level and mitochondrial PRS. All analyses were carried out using R programming language version 4.3.2. Graphs were made using the R package ggplot2 [44]. sPLS-DA was carried out by R package mixOmics (v6.26.0) [45].

## Results

The demographic and clinical characteristics of the cohort are summarized in Table 1. BMI at sampling and minimum BMI during AN were significantly lower, whereas the duration of amenorrhea was significantly longer in AN vs. AN-REC. The other characteristics were not significantly different between the two patient groups. Note that BMI at sampling was not significantly different between AN-REC and CTRL.

### Plasma GDF15

**Fig. 1:**
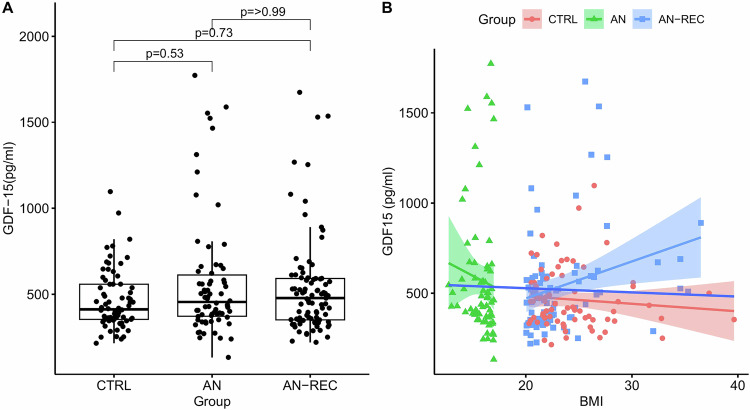
Growth and differentiation 15 (GDF15) concentration in plasma. **A** Plasma GDF15 in anorexia nervosa (AN), recovered anorexia nervosa (AN-REC), and healthy controls (CTRL). The median is shown as a straight line and the box denotes the interquartile range. **B** Correlations between plasma GDF15 and body mass index (BMI) in AN (−0.32, *p* = 0.0084, 95% CI [−0.53, −0.079]), AN-REC (0.29, *p* = 0.0058, 95% CI [0.082, 0.48]), CTRL (−0.15, *p* = 0.21, 95% CI [−0.38, 0.09]), and all groups combined (−0.031, *p* = 0.64, 95% CI [−0.16, 0.10]). The colored lines correspond to the correlation for all groups, and for the AN, AN-REC, and CTRL groups separately. The shaded area around each linear fit line represents a 95% confidence interval (CI).

Analysis of the genotype-corrected plasma concentration of GDF15 revealed no significant differences across groups (Fig. 1A). However, a subgroup of individuals had high concentrations of GDF15 (>800 pg/ml), with a significantly larger proportion found in the AN (*n* = 9) and AN-REC groups (*n* = 10) compared to CTRL (*n* = 2) (*p* = 0.038). Plasma GDF15 levels were negatively correlated with BMI in the AN group (*p* = 0.0084), while a positive correlation was found in the AN-REC group (*p* = 0.0058), (Fig. 1B). Inter-assay variability ranged between 13–15%.

### Plasma leptin and GDF15

**Fig. 2:**
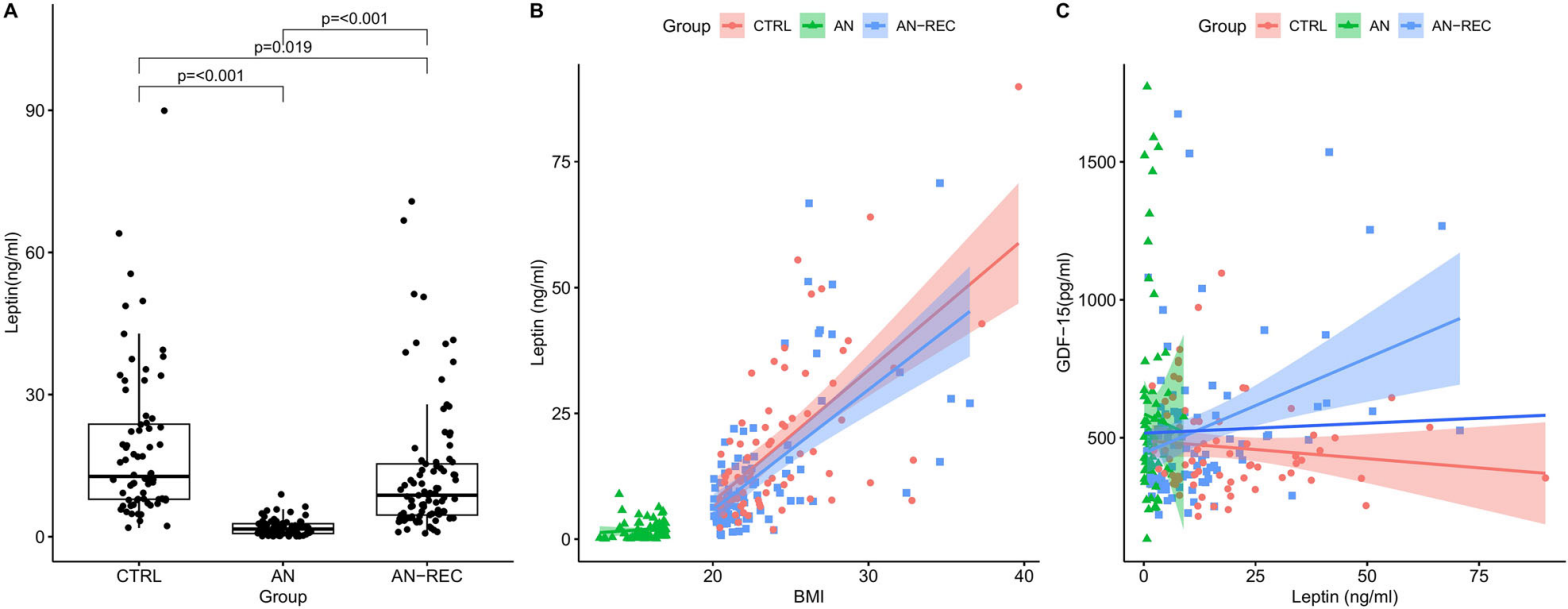
Leptin concentration in plasma. **A** Plasma leptin in anorexia nervosa (AN), recovered anorexia nervosa (ANREC), and healthy controls (CTRL). The median is shown as a straight line and the box denotes the interquartile range. **B** Correlations between plasma leptin and BMI in AN (0.28, *p* = 0.021, 95% CI [0.037, 0.49]), AN-REC (0.59, *p* = 1.94e–09, 95% CI [0.43, 0.71]), CTRL (0.55, *p* = 5.54e–07, 95% CI [0.36, 0.70]), and all groups combined (0.81, *p* 2.20e–16, 95% CI [0.75, 0.85]). **C** Correlations between plasma GDF15 and leptin in AN (−0.053, *p* = 0.67, 95% CI [−0.30, 0.20]), AN-REC (0.24, *p* = 0.028, 95% CI [0.020, 0.43]), CTRL (−0.16, *p* = 0.18, 95% CI [−0.39, 0.08]), and all groups combined (−0.013, *p* = 0.85, 95% CI [−0.15, 0.12]). The colored lines correspond to the correlation for all groups, and for the AN, AN-REC, and CTRL groups separately. The shaded area around each linear fit line represents a 95% confidence interval.

The plasma concentration of leptin was significantly reduced in AN and AN-REC compared with healthy controls, despite no difference in mean BMI at the time of sampling between the latter two groups. The plasma concentration of leptin in AN-REC was significantly higher than in the AN group (*p* < 0.001) (Fig. 2A) even after controlling for BMI (*p* = 0.01). Plasma leptin concentration was significantly correlated with BMI in all groups (Fig. 2B). Furthermore, plasma GDF15 was positively correlated with leptin concentration only in the AN-REC group (*p* = 0.028) (Fig. 2C).

### Plasma markers of inflammatory processes or cellular stress and GDF15

**Fig. 3:**
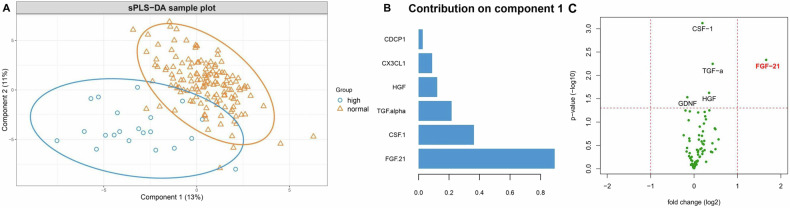
Plasma immune markers. **A** Sparse partial least squares discriminant analysis (sPLS-DA) sample plot of the 74 immune markers based on high GDF15 and normal GDF15 group with 95% confidence ellipses. Variances retained by the first two components are reported. **B** The loading plot represents the absolute values of the loading vectors for markers selected on Component 1 of the sPLS-DA model. **C** Volcano plot showing fold difference between high GDF15 group and normal GDF15 group. The x-axis represents fold change (log2), and the y-axis represents p-value (−log10). The data point representing FGF-21 is labeled (*p* = 0.0047). CDCP1 CUB domain-containing protein 1, CSF1 colony stimulating factor 1, FGF21 fibroblast growth factor 21, GDF15 growth and differentiation factor 15, HGF hepatocyte growth factor, TGF-a Transforming Growth Factor alpha.

A preliminary unsupervised PCA identified no major multi-dimensional outliers but also showed no separation between the group with high GDF15 concentration and the others (Supplementary Fig. 1). sPLS-DA revealed discrimination between the profiles of markers related to inflammatory processes or cellular stress of the two groups (Fig. 3A). The tuned sPLS-DA model (balanced error rate = 0.21), yielded by component and variable selection, consisted of two components, of which the first one best separated the group with high GDF15 from the rest by explaining 13% of the variance in GDF15 status. Six proteins constituted the first component, of which FGF21 was the major contributor (Fig. 3B). Furthermore, differential protein expression analysis identified that FGF21 was significantly higher in the high GDF15 group vs. the rest and was the only protein with a log2 fold change larger than 1.5 (Fig. 3C). Plasma FGF21 was significantly higher in the group of individuals with a high concentration of GDF15 in plasma (>800 pg/ml), compared with the rest of the individuals (*p* = 0.001). Group differences of other plasma markers identified by sPLS-DA and their correlation estimates with plasma GDF15 concentration are included in Supplementary Fig. 2.

As a proxy for recent physical exercise, we also reevaluated plasma levels of IL6 reported in our previous publication [38] and detected no significant difference between individuals with high GDF15 and the rest of the individuals. We also saw no significant correlation between plasma concentrations of IL-6 and GDF15 (Supplementary Fig. 2K, L).

### Mitochondrial PRS

**Fig. 4:**
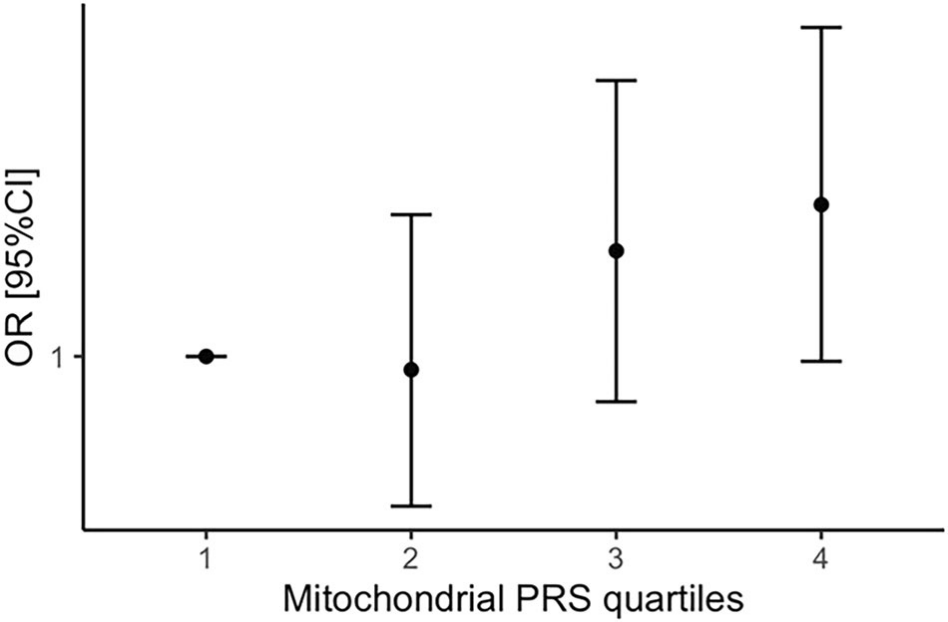
Odds Ratio (OR) with 95% confidence interval (95% CI) for anorexia nervosa (AN) by mitochondrial polygenic risk score (PRS) quartiles in ANGI-SE. The risk of AN is estimated using OR with 95% CI, with the lowest PRS quartile (1) as the reference group. The mitochondrial polygenic risk score (PRS) quartiles are defined as follows: 1 = low, 2 = mid-low, 3 = mid-high, and 4 = high.

Based on GDF15 and FGF21 in combination being used as markers of mitochondrial disorders [31] we evaluated PRS of mitochondrial function. Each standard deviation increase in the mitochondrial PRS was associated with 1.05 times greater odds of AN risk (95% CI [1.00, 1.09], *p* = 0.045). Individuals in the highest quartile of mitochondrial PRS had 1.13 times higher odds of AN compared to those in the lowest quartile (95% CI [1.00, 1.28]). There was a trend that the risk of AN increased across the mitochondrial PRS quartiles (Fig. 4), though larger sample sizes are needed to confirm these findings. However, no significant correlation was found between PRS scores and GDF15 level (*p* = 0.75) as well as FGF21 level (*p* = 0.29).

### GDF15 and FGF21 expression in *anx/anx* liver

**Fig. 5:**
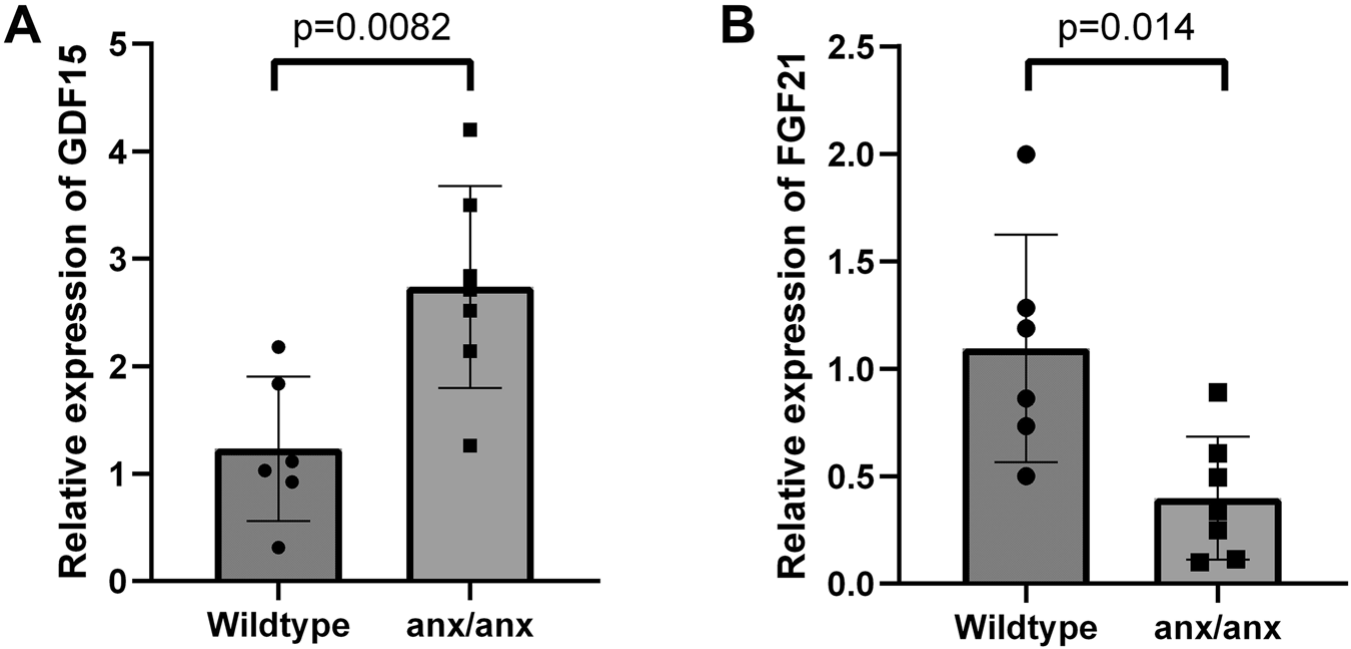
Liver expression of growth and differentiation factor 15 (GDF15) and fibroblast growth factor 21 (FGF21) in anorectic mice. **A** Relative expression levels of GDF15 and **B** FGF21 in *anx/anx* and wild-type female mice determined by qPCR. The bar charts present values as mean ± SD.

The relative expression of GDF15 in the liver of the anorectic *anx/anx* mouse was significantly higher compared with their wild-type siblings (Fig. 5A), while FGF21 was significantly lower (Fig. 5B).

### Plasma GDF15 and eating disorder characteristics

To further analyze GDF15 in relation to AN, we performed an exploratory analysis of plasma levels and eating disorder characteristics. Nominally significantly higher plasma concentrations of GDF15 were detected in the group of active AN patients who reported laxative use (*p* = 0.010, d = 0.91), self-induced vomiting (*p* = 0.017, d = 0.75), or compensatory exercise (*p* = 0.002, d = 0.74) compared with patients who reported no history of these behaviors (Supplementary Fig. 3). No significant differences were found in the group of AN-REC comparing those with and those without these behaviors, nor were any differences seen between those with vs without reported binge-eating behavior, or with vs without diuretic use in AN or AN-REC.

Supplementary Table 2 summarizes the clinical and eating disorder characteristics in the groups with elevated (>800 pg/ml) vs normal plasma GDF15(<800 pg/ml). Supplementary Table 3 shows the associations with these traits of the same two groups. Reported use of diuretics was nominally significantly associated with high plasma GDF15. Only two individuals in the full cohort, both within the group with high GDF15, were diagnosed with type 2 diabetes and cancer, thus no analysis of a potential association with these diagnoses was possible.

## Discussion

In contrast to smaller studies [5, 6], we did not detect any significant differences in plasma GDF15 concentration across the three groups; AN, AN-REC, and healthy controls. However, we found that a subgroup of women of a significantly larger number in the AN groups, have increased plasma GDF15 concentrations. We also observed a significant positive correlation between GDF15 and BMI, as well as with plasma leptin, in the AN-REC group. In the active AN group we only see a significant negative correlation between GDF15 and BMI, but not leptin, which may be explained by the near-zero concentrations of plasma leptin in this group. We speculate that the opposing correlations between GDF15 levels in plasma and BMI in AN (negative) vs AN-REC (positive) may be related to the history of starvation in the latter, since no correlation is seen in the control group with similar BMIs. When combining this plasma GDF15 and leptin data with data on 74 markers related to inflammatory processes or cellular stress from a previously published report from our group [38] in a sPLS-DA, we identified six markers that distinguished individuals with high plasma GDF15 from the rest of the individuals in the AN groups. Of these, FGF21 was defined as the most important contributor (see Supplementary Text). Plasma FGF21 was significantly higher only in the group of individuals with high GDF15. As mentioned above, FGF21 and GDF15 are elevated in animals [28, 29] as well as humans with mitochondrial dysfunction [30], and have been proposed to be used in combination as biomarkers to screen for pediatric mitochondrial disorders [31]. It is established that complex I dysfunction within the liver seen in e.g., Cockayne syndrome, gives rise to high concentrations of circulating GDF15 and FGF21 resulting in suppressed food intake, which is reversed by blocking GDF15 alone [46]. Thus, we speculate that mitochondrial dysfunction may be a factor in explaining the elevated plasma GDF15 seen in individuals predominantly belonging to the patient groups. In line with this, PRS calculated with SNPs within genes associated with mitochondrial function show a significant association with AN status in the full ANGI-SE cohort, suggesting a potential role of mitochondrial genes in AN risk and highlighting possible shared genetics between mitochondrial dysfunction and AN. The lack of correlations between the PRS scores and plasma levels of GDF15 and FGF21 were expected based on the in the context of PRS calculations small sample size used here. We also show significantly higher GDF15 expression in the liver from the *anx/anx* mouse compared to their wild-type siblings, whereas the liver expression of FGF21 was significantly lower in the *anx/anx* mouse, which was not in line with our hypothesis. We were however unable to measure the two proteins in *anx/anx* plasma, due to the small blood volume of these young and emaciated mice and thus cannot with certainty say that the plasma levels of FGF21 correspond to the level of expression of FGF21 in liver. The anorectic mouse model also displays dysfunction in complex I of the mitochondrial oxidative phosphorylation system [26], which has similarly been reported in a small cohort of patients with AN [27]. This indicates that further studies on mitochondrial dysfunction in AN should be prioritized.

FGF21 is another important energy metabolic regulator [47]. Animal studies show that the injection of FGF21 leads to elevated energy expenditure and adiponectin secretion from adipose tissue [48]. By simple diffusion, FGF21 can cross the blood-brain barrier and bind to its receptor expressed throughout the brain, particularly in the hypothalamus [49], which is the central control of feeding and energy expenditure. For example, FGF21 targets the lateral hypothalamus and acts on GABAergic neurons, which in turn stimulates thermogenesis and energy expenditure that leads to reduced weight gain [50]. FGF21 also activates glutamatergic neurons in the ventromedial hypothalamus to reduce sugar intake [51]. Moreover, subcutaneous administration of FGF21 increases the expression of the orexigenic neuropeptide AgRP and NPY in the arcuate nucleus of the hypothalamus [52]. In the context of AN, contradictory results on FGF21 levels in patients compared to controls have been reported. In a cohort of 11 patients and 12 controls, Pouneh et al. [53] reported significantly higher FGF21 in active AN patients, while Ivana et al. [54] showed reduced plasma FGF21 in AN patients in a cohort with 17 patients and controls. In Nilsson et al. we detected no difference in plasma FGF21 in active AN (*n* = 113), recovered AN (*n* = 113) and controls (*n* = 114) [36]. This despite that FGF21 expression is reported to be strongly induced by fasting [55] and protein restriction [56], and regulates energy homeostasis during starvation [55]. Based on this we were again surprised to see FGF21 expression not upregulated, but rather downregulated, in the *anx/anx* mouse liver.

Of note, lower plasma leptin levels were observed not only in AN but also in AN-REC compared to healthy controls, despite the latter two groups having similar mean BMI. Previous data on leptin in individuals recovered from AN have been mixed, while some studies have reported that serum leptin rises with weight recovery [57, 58] and even reaches values above those observed in controls matched for BMI [59], others report normal leptin in both cerebrospinal fluid and serum with long term recovery [60]. Thus, the potential role of leptin in AN recovery remains to be established.

As with leptin, GDF15 is related to physical activity. Although low leptin is speculated to play a role in the increased physical activity of AN [61], GDF15 is reported to markedly increase with intense exercise in humans as well as mice [19, 20], and we here detected increased plasma GDF15 in the group with active AN reporting compensatory exercise. However, we lack information on physical activity directly prior to sampling. As a proxy for recent intense exercise [62], we compared plasma concentration of the exercise-induced myokine IL6 in the group of individuals with high vs. normal plasma GDF15 but saw no differences. Thus, this indicates that the increased GDF15 seen in the group of individuals here is unlikely to be related to intense exercise prior to sampling.

In stratified analyses, we found that individuals with active AN who report compensatory behaviors, i.e., laxative use, self-induced vomiting, and as already mentioned compensatory exercise, have nominally significantly higher plasma GDF15 than individuals with active AN reporting no such behaviors. Purging behaviors were not associated with higher plasma GDF15 in AN-REC.

This is the to date largest study evaluating GDF15 levels in AN. The inclusion of samples from individuals recovered from AN, data from anorectic animals as well as genetic data gives strength to the study. A limitation of this study is that we did not have information about the state of the participants, in particular fasted vs fed, when sampling was done. However, little variation in GDF15 concentration has been reported with meals [63], fasting, and refeeding [64]. One study reported peak plasma GDF15 concentration after 48 h of severe caloric restriction [63], while other studies have shown no change in plasma GDF15 following 8 weeks or 6 months of low-calorie dietary plans, despite a total body weight loss of 11 and 13.5%, respectively [65, 66]. We also did not have information on the time of the day of sampling. But even if circulating levels of GDF15 vary in a diurnal pattern with 10% plus or minus [64], this should not account for the high plasma concentrations seen for some individuals in this study. With the exception of cancer and diabetic diagnoses, we were limited by having had no information on other factors/conditions that could influence GDF15 levels in plasma e.g., smoking [67] and medications. Lastly, due to the small blood volume of the young and anorectic *anx/anx* mouse we were restricted to measuring GDF15 expression in liver rather than GDF15 protein in plasma.

To conclude, we observed no differences in plasma GDF15 across the AN, AN-REC, or CTRL groups, but identified a subgroup of individuals almost exclusively within the two patient groups who have high plasma GDF15 concentrations. This leads us to hypothesize that, if our results are replicated, neutralizing GDF15 may have the potential to support appetite and aid in normalizing food intake in some individuals with AN. The group of individuals with high plasma GDF15 also had higher concentrations of FGF21 in plasma. FGF21 was the main distinguishing contributor of the group of individuals with high GDF15, which may suggest mitochondrial dysfunction in this group of individuals with current or past AN. Associations between mitochondrial PRS and AN risk further support the potential shared genetic basis between AN and mitochondrial dysfunction. In line with this, we report markedly increased GDF15 expression, while FGF21 surprisingly is reduced, in the liver from the anorectic *anx/anx* mouse previously reported to display mitochondrial dysfunction. Thus, our findings support the continued evaluation of mitochondrial function in AN, and clinical trials of GDF15 immunoneutralization in patients with AN and high levels of GDF15 are worth consideration.

## Supplementary information

Supplementary material

Supplementary Table 1

## Data Availability

Data supporting the findings of this study can be made available from the corresponding authors upon request.
