# Supplementary material for: Elevated plasma GDF15 combined with FGF21 suggests mitochondrial dysfunction in a subgroup of anorexia nervosa patients
Source: Transl Psychiatry. 2025 Jun 25;15:215. doi: 10.1038/s41398-025-03425-0 (PMC12198413; doi:10.1038/s41398-025-03425-0)
Supplement: Supplementary file 1 — Supplementary material [file 41398_2025_3425_MOESM1_ESM.docx]

**Supplementary material:**

- **Supplementary methods**
- **Supplementary tables 1-3**
- **Supplementary figure 1-3**

**Supplementary methods**

**sPLS-DA**

The identification of FGF21 as the top contributor of the immune activation markers for separating high GDF15 and normal GDF15 groups is the result of a finely-tuned sPLS-DA model. The advantage of sPLS-DA over other multivariate and feature selection techniques is that it is a supervised dimension reduction methodology that performs variable selection and classification in a one-step procedure. As long as a dataset has enough features, sPLS-DA functions are able to separate the dataset according to predefined classes; however, it is prone to overfitting and most of the separating hyperplanes may be just “noise” [^52^](#_ENREF_52). Therefore, it is crucial to select the numbers of components and variables in the model and adjust by cross-validation to reach best performance. In this study, the initial model starts with ten components and includes all 74 immune activation markers. Considering the unbalanced nature of dataset, only 21 individuals in the high GDF15 group compared with 138 in the other group, cross-validation of five-fold was used to balance bias with variance. Model performance was determined by minimal balanced error rate, which accounts for average of classification errors on each class. The final model consisted of two components and only six markers were selected in the first component, FGF21 being the marker with the largest absolute value of loading vector, indicating a greater contribution. This is supported by other feature selection techniques such as Lasso regression, which yielded similar results (data not shown).

**Supplementary table 1.** Genes related to mitochondrial function used in polygenic risk score calculations - attached as an excel file.

**Supplementary table 2**. Age, BMI, years since AN onset, and eating disorder behaviors of the study participants by GDF15 concentration in plasma.

| **Characteristics** | **GDF15 >800 pg/ml** | **GDF15 <800 pg/ml** |
| --- | --- | --- |
| **n** | 21 | 138 |
| **Age at sample (years)** | 30 | 26 |
| **(median [IQR])** | (25.5-35.0) | (24.0-30.0) |
| **Age of first AN onset (years)** | 16 | 16 |
| **(median [IQR])** | (14.5-19.5) | (14.0-19.0) |
| **BMI at sample (kg/m^2^)** | 20 | 20.4 |
| **(median [IQR])** | (15.5-25.8) | (16.4-22.0) |
| **Minimum BMI during AN (kg/m^2^)** | 14.7 | 14.7 |
| **(median [IQR])** | (13.1-17.5) | (13.6-16.6) |
| **Years since AN onset** | 11 | 10 |
| **(median [IQR])** | (6.0-18.0) | (6.0-14.0) |
| **Length of amenorrhea (years)** | 3 | 2 |
| **(median [IQR])** | (2.5-5.0) | (1.0-5.5) |
| **Length of vomiting (n [%])** |  |  |
| **Longer than 1 year** | 8 (72.7%) | 38 (50.7%) |
| **Less than 1 year** | 3 (27.3%) | 37 (49.3%) |
| **Frequency of vomiting (n [%])** |  |  |
| **Daily** | 6 (50%) | 33 (40.7%) |
| **Less than once a day** | 6 (50%) | 48 (59.3%) |

AN, anorexia nervosa; BMI, body mass index; GDF15; growth and differentiation factor 15; IQR, interquartile range.

**Supplementary table 3**. Contingency table of associations between high plasma GDF15 concentration and eating disorder behaviors in AN and AN-REC.

|  | **Binge eating** | **no binge eating** | **total** |
| --- | --- | --- | --- |
| **GDF15 >800 pg/ml** | 10 | 10 | 20 |
| **GDF15 <800 pg/ml** | 75 | 51 | 126 |
| **Total** | 85 | 61 | 146 |
| **p-value, φ** | p =0.58, φ = 0.046 | |  |
|  | **laxative use** | **no laxative use** | **total** |
| **GDF15 >800pg/ml** | 5 | 16 | 21 |
| **GDF15 <800 pg/ml** | 24 | 111 | 135 |
| **total** | 29 | 127 | 156 |
| **p-value, OR** | p = 0.55, OR = 1.44 | |  |
|  | **self ind. vomiting** | **no vomiting** | **total** |
| **GDF15 >800pg/ml** | 13 | 8 | 21 |
| **GDF15 <800 pg/ml** | 62 | 73 | 135 |
| **total** | 75 | 81 | 156 |
| **p-value, φ** | p = 0.26, φ = 0.09 | |  |
|  | **comp. exercise** | **no comp. exercise** | **total** |
| **GDF15 >800pg/ml** | 19 | 2 | 21 |
| **GDF15 <800 pg/ml** | 116 | 19 | 135 |
| **total** | 135 | 21 | 156 |
| **p-value, OR** | p = 0.74, OR = 1.55 | |  |
|  | **diuretics use** | **no diuretics use** | **total** |
| **GDF15 >800pg/ml** | 5 | 16 | 21 |
| **GDF15 <800 pg/ml** | 9 | 126 | 135 |
| **total** | 14 | 142 | 156 |
| **p-value, OR** | p = 0.024, OR = 4.31 | |  |

comp.exercise, compensatory exercise; GDF15, growth and differentiation factor 15; self ind. vomiting, self-induced vomiting.

**
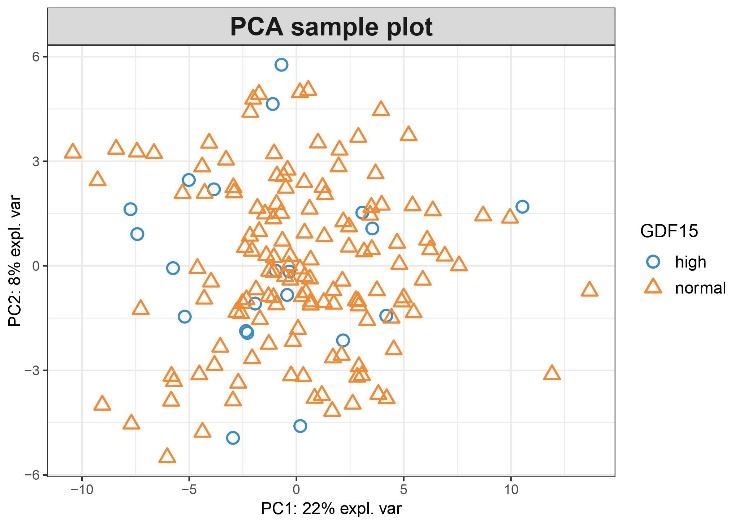
**

**Supplementary figure 1.** Principle component analysis (PCA) sample plot based on high growth and differentiation factor 15 (GDF15) concentration group and normal GDF15 concentration group. Variances retained by the first two principal components are reported.


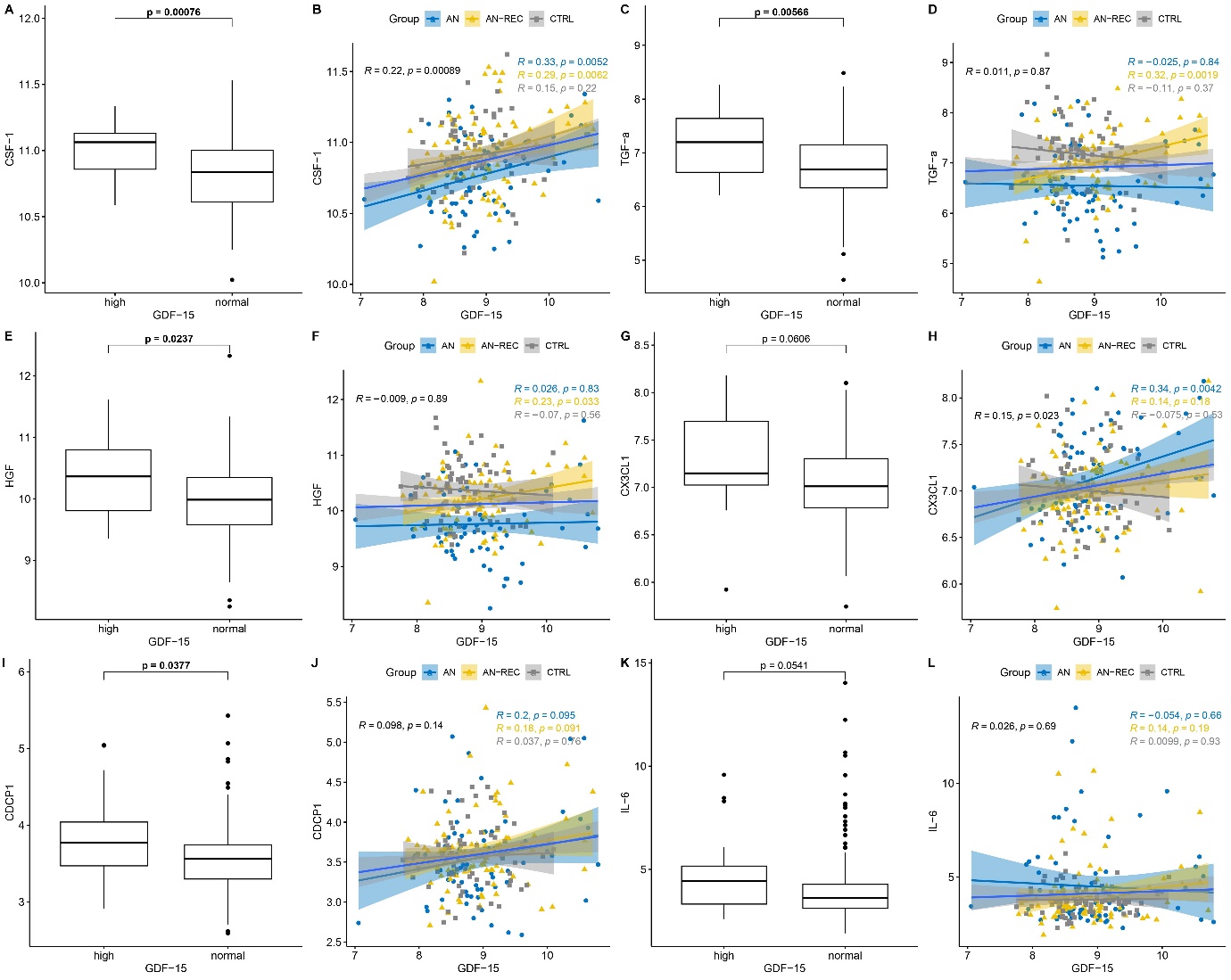


**Supplementary figure 2.** Box plots illustrate differences in markers related to inflammatory processes or cellular stress (CDCP1, CUB domain-containing protein 1; CSF1, colony-stimulating factor 1; FGF21, fibroblast growth factor 21; GDF15, growth and differentiation factor 21; HGF, hepatocyte growth factor; TGF alpha, Transforming Growth Factor alpha) identified by sparse partial least squares discriminant analysis (sPLS-DA) (A, C, E, G, I), and interleukin 6 (IL-6) (K) comparing individuals with high GDF15 (> 800 pg/ml) with the rest of the individuals (< 800 pg/ml). The median is shown as a straight line and the box denotes the interquartile range. Graphs show Spearman correlations between GDF15 and plasma concentrations of sPLS-DA identified markers (B, D, F, H, J), and IL-6 (L). The colored lines correspond to the correlation for all groups, and for the anorexia nervosa (AN), recovered anorexia nervosa (AN-REC), and healthy control (CTRL) groups separately. The shaded area around each linear fit line represents a 95% confidence interval (CI).


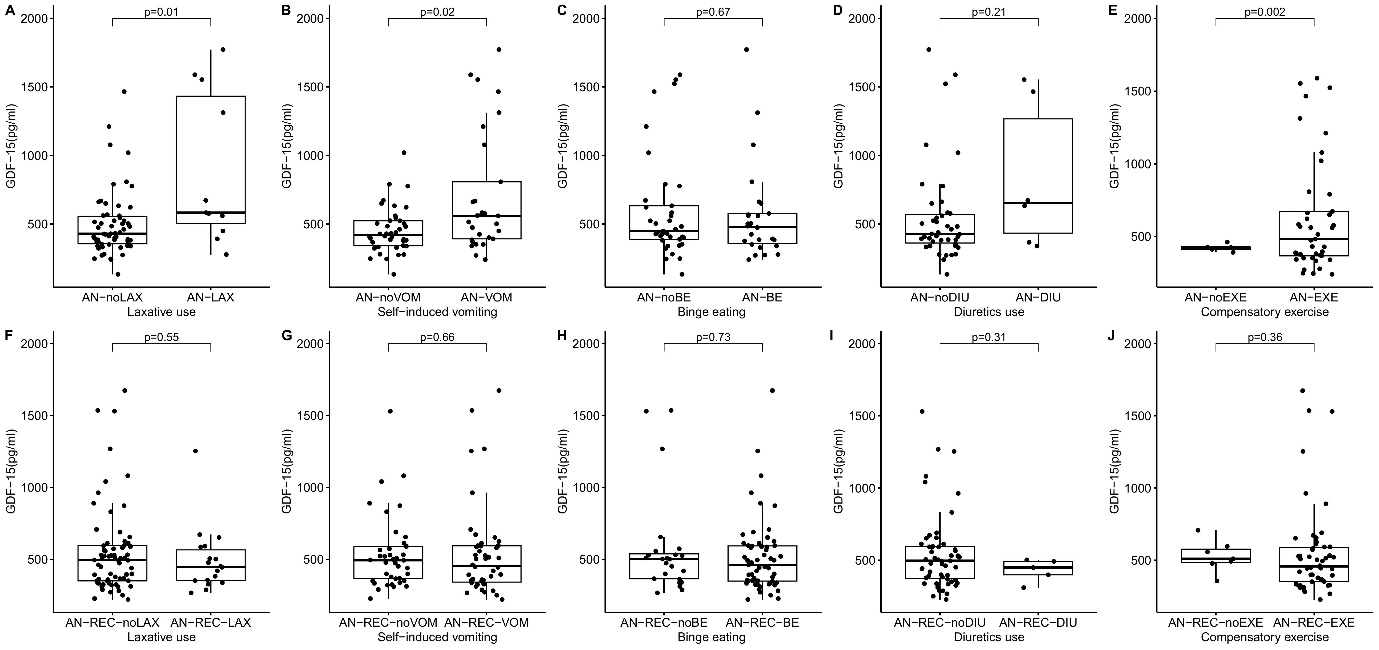


**Supplementary figure 3.** Growth and differentiation factor 15 (GDF15) concentration in plasma from individuals with anorexia nervosa (AN) (A, C, E, G, I) or recovered from anorexia nervosa (AN-REC) (B, D, F, H, J) with or without a history of laxative use (LAX) (A, B), self-induced vomiting (VOM) (C, D), binge eating (BE) (E, F), diuretics use (DIU)(G, H), and compensatory exercise (EXE) (I, J). The median is shown as a straight line and the box denotes the interquartile range.
